# Supplementary material for: The antigenic landscape of human influenza N2 neuraminidases from 2009 until 2017
Source: eLife. 2024 May 28;12:RP90782. doi: 10.7554/eLife.90782 (PMC11132685; doi:10.7554/eLife.90782)
Supplement: Supplementary file 1. [file elife-90782-supp1.docx]

**Specific activity of the recombinant NACHOs.**

| H3N2 strain NA source | **Specific Activity nmole/min.µg** |
| --- | --- |
| A/Stockholm/32/2014 | 9,62 |
| A/Switzerland/9715293/2013 | 6,52 |
| A/Hong Kong/4801/2014 | 4,13 |
| A/Paris/2379/2014 | 2,42 |
| A/Texas/50/2012 | 5,21 |
| A/Stockholm/14/2012 | 2,42 |
| A/Tehran/996/2012 | 8,46 |
| A/Victoria/361/2011 | 2,71 |
| A/Newcastle/67/2016 | 3,22 |
| A/Estonia/91621/2015 | 5,3 |
| A/Ankara/2396/2015 | 2,62 |
| A/Stockholm/15/2014 | 1,14 |
| A/Dakar/14/2014 | 2,37 |
| A/PortoAlegre/Lacenrs-2376/2014 | 2,55 |
| A/Utah/11/2011 | 1,40 |
| A/Hokkaido/IOH079/2011 | 0,84 |
| A/Gambia/G0071436/2012 | 1,60 |
| A/Hanoi/Eli15597/2015 | 3,29 |
| A/XinJiang-Tianshan/1411/2012 | 0,27 |
| A/Perth/16/2009nib-64 | 5,3 |
| A/Tasmania/1018/2015 | 27,48 |
| A/NewCaledonia/23/2016 | 8,55 |
| A/Alaska/251/2015 | 4,29 |
| A/Hatay/4990/2016 | 5,83 |
| A/Tennessee/18/2017 | 5,99 |
| A/Singapore/Infimh160019/2016 | 8,01 |
| A/Brisbane/273/2016 | 6,94 |
| A/Poland/19b/2017 | 5,81 |
| A/Wisconsin/16/2015 | 1,40 |
| A/Indiana/08/2011 | 4,13 |
| A/Hong-Kong/3089/2017 | 6,17 |
| A/Nagano/2153/2017 | 2,29 |
| A/Sweden/3/2017 | 2,76 |
| A/Catalonia/9503s/2017 | 1,31 |
| A/Moramanga/1907/2017 | 2,09 |
| A/Heilongjiang_Xiangyan/1134/2011 | 6,61 |
| A/Helsinki/823/2013 | 6,53 |
| A/Helsinki/941/2013 | 7,02 |
| A/Ohio/13/2017 | 2,59 |
| A/Ohio/62/2012 | 4,28 |
| A/Ontario/RV3236/2016 | 3,89 |
| A/Indiana/18/2017 | 4,27 |
| A/Minnesota/11/2010 | 4,25 |
