# Supplementary material for: The antigenic landscape of human influenza N2 neuraminidases from 2009 until 2017
Source: eLife. 2024 May 28;12:RP90782. doi: 10.7554/eLife.90782 (PMC11132685; doi:10.7554/eLife.90782)
Supplement: Supplementary file 2. [file elife-90782-supp2.docx]

**H6N2 reassortant viruses used in the ELLA assay.**

| H6Nx reassortant(*) | Titer (PFU/ml) | dilution to 70% max activity (ELLA) | K_M_ (MUNANA) |
| --- | --- | --- | --- |
| A/Ohio/62/2012 (Ohi12) | 2.00E+08 | 2269.7 | 20.7 |
| A/Indiana/08/2011 (Ind11) | 2.50E+08 | 750.9 | 40.1 |
| A/Ohio/13/2017 (Ohi17) | 3.00E+08 | 521.5 | 33.5 |
| A/Ontario/Rv3236/2016 (Ont16) | 1.75E+08 | 504.1 | 31.5 |
| A/Minnesota/11/2010 (Min10) | 2.00E+08 | 1031.4 | 41.3 |
| A/Helsinki/823/2013 (Hel823) | 1.50E+08 | 155.2 | 45.9 |
| A/Helsinki/941/2013 (Hel941) | 3.50E+08 | 341.6 | 45.3 |
| A/Nagano/2153/2017 (Nag17) | 2.50E+08 | 213.6 | 97.8 |
| A/Singapore/Infimh-16-0019/2016 (Sin16) | 6.50E+07 | 257.1 | 93.3 |
| A/Hong_Kong/3089/2017 (HK17) | 2.00E+08 | 301.6 | 85.2 |
| A/Sweden/3/2017 (Swe17) | 6.00E+08 | 97 | 71.5 |
| A/Moramanga/1907/2017 (Mor17) | 1.40E+08 | 151.4 | 56.8 |
| A/Kansas/14/2017 (Kan17) | 2.50E+08 | 249 | 108.7 |
| A/Wisconsin/16/2015 (Wis15) | 2.00E+08 | 63.3 | 79.6 |
| A/Gambia/G0071436/2012 (Gam12) | 2.50E+08 | 331.5 | 63.6 |
| A/Heilongjiang-Xiangyang/1134/2011 (Hei11) | 2.10E+08 | 195.6 | 31.1 |
| A/Perth/16/2009 (Per09) | 3.20E+08 | 630.8 | 56.4 |
| A/Newcastle/67/2016 (NCas16) | 1.50E+08 | 150.4 | 48.5 |
| A/Tasmania/1018/2015 (Tas15) | 1.70E+08 | 139.5 | 54.8 |
| A/Hanoi/Eli15597/2015 (Han15) | 1.40E+08 | 94.6 | 82.2 |
| A/Alaska/251/2015 (Ala15) | 4.50E+08 | 93.7 | 144.8 |
| A/Estonia/91621/2015 (Est15) | 2.00E+08 | 281.9 | 62.1 |
| A/Victoria/361/2011 (Vic11) | 3.00E+08 | 570.3 | 51.1 |
| A/Utah/11/2011 (Uta11) | 2.50E+08 | 356.3 | 51.9 |
| A/Switzerland/9715293/2013 (Swi13) | 2.70E+08 | 160.8 | 64 |
| A/Hong Kong/4801/2014 (HK14) | 4.00E+07 | 106.3 | 54.7 |
| A/Texas/50/2012 (Tex12) | 2.00E+08 | 276.6 | 45.1 |

*Name of the virus from which the N2 NA in H6N2 reassortant virus was derived. The short name as used in the manuscript text is mentioned between brackets.
