# Supplementary material for: The antigenic landscape of human influenza N2 neuraminidases from 2009 until 2017
Source: eLife. 2024 May 28;12:RP90782. doi: 10.7554/eLife.90782 (PMC11132685; doi:10.7554/eLife.90782)
Supplement: Supplementary file 3. [file elife-90782-supp3.docx]

**NAI titers against H6N2 viruses of ferret sera obtained after the boost (Part I of II).**

|  | |  |  |  |  |  |  |  |  |  |  |  |  |  |
| --- | --- | --- | --- | --- | --- | --- | --- | --- | --- | --- | --- | --- | --- | --- |
| **Sera** | **Swi13** | | **HK14** | **Tex12** | **Vic11** | **Ncas16** | **Est15** | **Uta11** | **Gam12** | **Han15** | **Hei11** | **Per09** | **Tas15** | **Ala15** |
| **A/Switzerland/9715293/2013** | **9333.0** | | 6833.5 | 10301.6 | 2442.3 | 1489.8 | 5530.4 | 1460.6 | 3489.9 | 5253.8 | 1510.9 | 1899.6 | 1649.9 | 1812.3 |
| **A/Hong_Kong/4801/2014** | 7527.5 | | **10566.4** | 14983.0 | 2559.1 | 4579.4 | 7944.5 | 3590.3 | 7065.0 | 4798.3 | 1971.2 | 2522.9 | 3842.2 | 4980.9 |
| **A/Texas/50/2012** | 10751.3 | | 10397.6 | **8843.5** | 2089.6 | 740.9 | 3821.0 | 902.1 | 1519.1 | 1453.1 | 1112.9 | 2506.5 | 835.8 | 1490.8 |
| **A/Victoria/361/2011(Ivr-165)** | 16804.7 | | 11568.3 | 11892.0 | **3863.7** | 1868.9 | 3480.4 | 2250.0 | 670.9 | 3963.3 | 1379.1 | 2888.7 | 883.1 | 1531.9 |
| **A/Newcastle/67/2016** | 7647.2 | | 9702.6 | 5244.0 | 1056.9 | **4218.8** | 2295.9 | 5477.0 | 821.2 | 1515.9 | 1254.3 | 1304.0 | 1253.1 | 1021.6 |
| **A/Estonia/91621/2015** | 1277.1 | | 1379.5 | 911.1 | 387.1 | 290.5 | **2832.9** | 277.9 | 335.3 | 225.6 | 272.5 | 193.3 | 217.2 | 70.9 |
| **A/Utah/11/2011** | 6121.8 | | 6591.4 | 6253.2 | 3392.5 | 1570.0 | 3727.5 | **2893.7** | 1294.7 | 2496.3 | 4050.7 | 2196.7 | 376.0 | 869.2 |
| **A/Gambia/G0071436/2012** | 5513.7 | | 6192.0 | 5040.0 | 1226.5 | 1492.3 | 6342.9 | 772.9 | **730.2** | 863.5 | 1810.2 | 1399.4 | 836.9 | 888.0 |
| **A/Hanoi/Eli15597/2015** | 4635.2 | | 8150.5 | 6232.1 | 872.7 | 1617.0 | 7192.3 | 1023.4 | 1343.3 | **1822.0** | 1969.9 | 1168.3 | 1307.0 | 2383.4 |
| **A/Heilongjiang-Xiangyang/1134/2011** | 3506.3 | | 3480.2 | 5123.0 | 3514.3 | 356.0 | 976.7 | 1657.1 | 245.9 | 557.9 | **7094.8** | 2056.7 | 168.3 | 658.3 |
| **A/Perth/16/2009nib-64** | 4961.8 | | 13872.0 | 8102.7 | 2771.2 | 630.3 | 3502.5 | 2644.5 | 1079.5 | 441.6 | 1663.8 | **2458.4** | 418.4 | 748.3 |
| **A/Tasmania/1018/2015** | 6503.6 | | 7894.2 | 5204.6 | 1298.1 | 2335.3 | 2927.7 | 1349.1 | 6451.6 | 2374.2 | 709.1 | 2659.2 | **1150.5** | 977.3 |
| **A/Alaska/251/2015** | 7291.7 | | 8216.5 | 7274.8 | 1247.6 | 1050.4 | 7445.9 | 1975.1 | 348.6 | 1070.2 | 1828.5 | 1142.7 | 2300.4 | **3418.1** |
| **A/Singapore/Infimh-16-0019/2016** | 2564.5 | | 3869.9 | 3206.3 | 1119.2 | 1038.5 | 732.7 | 545.2 | 252.3 | 1308.7 | 196.5 | 328.1 | 399.6 | 778.4 |
| **A/Wisconsin/16/2015** | 6157.8 | | 6249.7 | 3088.6 | 693.4 | 998.2 | 1259.1 | 383.6 | 73.1 | 595.7 | 362.7 | 415.4 | 442.8 | 723.5 |
| **A/Hong_Kong/3089/2017** | 713.6 | | 1372.5 | 1290.5 | 391.8 | 312.4 | 670.9 | 231.0 | 144.6 | 161.5 | 143.3 | 88.2 | 317.5 | 702.9 |
| **A/Nagano/2153/2017** | 784.6 | | 798.8 | 567.6 | 102.6 | 215.5 | 530.1 | 158.5 | 97.5 | 182.8 | 145.2 | 108.7 | 172.4 | 332.8 |
| **A/Sweden/3/2017** | 2250.6 | | 1249.6 | 2688.6 | 1818.4 | 785.2 | 443.3 | 632.2 | 726.1 | 3344.3 | 553.2 | 512.0 | 910.7 | 2153.6 |
| **A/Moramanga/1907/2017** | 1126.6 | | 1371.0 | 720.8 | 205.3 | 185.9 | 1073.8 | 339.2 | 212.5 | 335.6 | 228.0 | 201.0 | 396.1 | 603.7 |
| **A/Helsinki/823/2013** | 1130.4 | | 1408.4 | 1382.9 | 491.8 | 77.1 | 1930.9 | 324.0 | 141.1 | 133.6 | 431.4 | 289.4 | 90.0 | 106.9 |
| **A/Helsinki/941/2013** | 391.1 | | 379.7 | 662.2 | 345.4 | 19.2 | 837.0 | 227.2 | 32.7 | 25.7 | 72.6 | 111.1 | 23.7 | 58.9 |
| **A/Ohio/13/2017** | 396.6 | | 554.6 | 500.2 | 247.4 | 56.6 | 377.9 | 185.1 | 112.0 | 75.2 | 168.4 | 219.8 | 80.1 | <20 |
| **A/Ohio/62/2012** | 445.7 | | 644.5 | 694.0 | 157.0 | 33.1 | 1024.6 | 207.9 | 37.9 | 77.9 | 98.5 | 152.6 | 86.9 | 22.5 |
| **A/Ontario/Rv3236/2016** | 54.0 | | 99.2 | 101.7 | 218.0 | <20 | 51.7 | 43.8 | <20 | <20 | <20 | 36.9 | 41.1 | <20 |
| **A/Indiana/08/2011** | 34.4 | | 72.5 | 110.4 | 177.3 | 22.4 | 43.1 | 46.8 | 31.4 | <20 | 40.6 | 36.4 | <20 | <20 |
| **A/Minnesota/11/2010** | 59.4 | | 146.7 | 91.6 | <20 | <20 | 44.0 | 39.0 | <20 | <20 | 41.1 | 30.8 | <20 | <20 |

**Table S3. NAI titers against H6N2 viruses of ferret sera obtained after the boost (Part II of II).**

| **Sera** | **Sin16** | **Wis15** | **HK17** | **Nag17** | **Swe17** | **Mor17** | **Hel823** | **Hel941** | **Ohi17** | **Ohi12** | **Ont16** | **Ind11** | **Min11** |
| --- | --- | --- | --- | --- | --- | --- | --- | --- | --- | --- | --- | --- | --- |
| **A/Switzerland/9715293/2013** | 1291.6 | 939.6 | 1097.8 | 210.5 | 514.9 | 460.1 | 160.2 | 706.4 | 422.8 | 1072.5 | 354.5 | 227.5 | 302.5 |
| **A/Hong_Kong/4801/2014** | 636.5 | 780.7 | 1312.1 | 30.8 | 178.6 | 231.7 | 55.5 | 229.9 | 221.4 | 143.0 | 122.2 | 93.4 | 307.2 |
| **A/Texas/50/2012** | 436.8 | 556.3 | 462.7 | <20 | 242.5 | 288.5 | 53.2 | 656.4 | 73.0 | 295.0 | 482.4 | <20 | 149.6 |
| **A/Victoria/361/2011(Ivr-165)** | 568.2 | 1881.1 | 1227.3 | 108.5 | 293.8 | 407.7 | 79.3 | 1203.0 | 290.1 | 561.0 | 793.8 | 125.4 | 393.1 |
| **A/Newcastle/67/2016** | 192.6 | 246.4 | 411.3 | 18.3 | 70.8 | 125.6 | 206.8 | 259.5 | 893.7 | 1553.0 | 1089.8 | <20 | 1619.5 |
| **A/Estonia/91621/2015** | 133.6 | 137.5 | 252.2 | <20 | 78.5 | <20 | 112.6 | 76.3 | 96.2 | 268.4 | 110.6 | 38.7 | 83.6 |
| **A/Utah/11/2011** | 212.4 | 753.0 | 215.9 | 41.4 | 138.5 | 265.5 | 100.4 | 549.1 | 208.9 | 1246.7 | 922.8 | 240.0 | 466.7 |
| **A/Gambia/G0071436/2012** | 390.8 | 532.3 | 694.8 | 37.3 | 174.6 | 139.2 | 296.9 | 470.4 | 233.7 | 154.4 | 423.5 | 157.8 | 709.3 |
| **A/Hanoi/Eli15597/2015** | 225.9 | 629.8 | 592.7 | 152.6 | 318.2 | 174.4 | 216.5 | 209.5 | 615.2 | 207.1 | 556.5 | 1205.8 | 669.2 |
| **A/Heilongjiang-Xiangyang/1134/2011** | 79.9 | 257.3 | 65.1 | <20 | 77.1 | 34.9 | 475.2 | 76.0 | 236.7 | 1330.2 | 70.6 | 48.1 | 127.2 |
| **A/Perth/16/2009nib-64** | 263.1 | 535.5 | 249.8 | <20 | 102.5 | 103.7 | 552.5 | 519.6 | 250.2 | 418.2 | 81.2 | 113.6 | 161.1 |
| **A/Tasmania/1018/2015** | 714.6 | 342.3 | 1149.8 | 178.8 | 321.5 | 551.2 | 363.1 | 1440.4 | 407.4 | 2702.2 | 819.1 | 463.8 | 689.9 |
| **A/Alaska/251/2015** | 655.6 | 1029.8 | 579.3 | 451.3 | 764.5 | 396.5 | 111.7 | 468.9 | <20 | 72.8 | 88.1 | <20 | 22.4 |
| **A/Singapore/Infimh-16-0019/2016** | **3746.2** | 1772.7 | 7210.9 | 1390.6 | 1637.5 | 1258.2 | 151.7 | 2123.3 | 94.6 | 282.6 | 68.0 | <20 | 122.3 |
| **A/Wisconsin/16/2015** | 772.0 | **5850.3** | 678.1 | 373.4 | 680.7 | 420.1 | <20 | 134.2 | <20 | 74.7 | <20 | <20 | <20 |
| **A/Hong_Kong/3089/2017** | 1534.5 | 708.7 | **4119.2** | 762.8 | 2114.3 | 744.7 | 297.3 | 324.9 | <20 | <20 | <20 | <20 | <20 |
| **A/Nagano/2153/2017** | 501.4 | 157.0 | 1131.7 | **960.8** | 258.6 | 313.2 | 94.3 | 115.9 | 85.6 | 136.8 | <20 | <20 | 54.6 |
| **A/Sweden/3/2017** | 5336.2 | 1167.5 | 7099.8 | 1132.6 | **8452.0** | 4595.1 | 506.4 | 2310.2 | 133.0 | 199.5 | 323.9 | 27.2 | 58.5 |
| **A/Moramanga/1907/2017** | 1060.6 | 273.0 | 3033.6 | 258.9 | 1286.0 | **6330.8** | 75.6 | 143.2 | 57.3 | 106.0 | 96.7 | <20 | 70.4 |
| **A/Helsinki/823/2013** | 519.3 | 215.9 | 745.1 | <20 | 326.8 | 113.1 | **5933.4** | 11949.0 | 33.1 | 87.9 | 85.5 | 32.4 | 46.8 |
| **A/Helsinki/941/2013** | 257.3 | 79.5 | 156.3 | <20 | 153.0 | <20 | 3729.4 | **17816.2** | <20 | 64.1 | <20 | <20 | <20 |
| **A/Ohio/13/2017** | 28.9 | <20 | <20 | <20 | <20 | <20 | 221.2 | 137.9 | **2567.9** | 28683.0 | 4819.3 | 781.1 | 4943.5 |
| **A/Ohio/62/2012** | <20 | <20 | <20 | <20 | <20 | <20 | 77.1 | 70.1 | 5592.0 | **3811.0** | 397.1 | 281.1 | 921.4 |
| **A/Ontario/Rv3236/2016** | <20 | <20 | <20 | <20 | <20 | <20 | 22.4 | 21.3 | 194.8 | 1500.5 | **6167.6** | 159.3 | 846.2 |
| **A/Indiana/08/2011** | <20 | <20 | <20 | <20 | <20 | <20 | <20 | <20 | 146.4 | 3741.8 | 486.8 | **2910.9** | 2498.3 |
| **A/Minnesota/11/2010** | <20 | <20 | <20 | <20 | <20 | <20 | 55.0 | <20 | 202.3 | 509.1 | 726.6 | 380.2 | **4375.6** |
